# Dramatic resolution of vitreous hemorrhage after an intravitreal injection of dobesilate

**DOI:** 10.1186/s40779-015-0050-5

**Published:** 2015-09-08

**Authors:** Pedro Cuevas, Luis Antonio Outeiriño, Carlos Azanza, Javier Angulo, Guillermo Giménez-Gallego

**Affiliations:** Facultad de Medicina, Universidad Alfonso X, Madrid, Spain; Departamento de Oftalmología, Hospital de Día Pío XII, Madrid, Spain; Servicio de Histología, Departamento, de Investigación, IRYCIS, Hospital Universitario Ramón y Cajal, Madrid, Spain; Departamento de Estructura y Función de Proteínas, Centro de Investigaciones Biológicas, CSIC, Madrid, Spain

**Keywords:** Proliferative diabetic retinopathy, Vitreous hemorrhage, Dobesilate

## Abstract

Vitreous hemorrhages are important clinical manifestations of proliferative diabetic retinopathy. Non-cleared vitreous hemorrhages could lead to hemosiderosis bulbi and glaucoma. Here, we describe the case of a type 2 diabetic patient presenting anterior segment and vitreous hemorrhages that resolved three days after treatment with a single intravitreal injection of dobesilate.

## Background

Vitreous hemorrhages are an important clinical manifestation of proliferative diabetic retinopathy. A vitreous hemorrhage may spontaneously get re-absorbed over time in some cases. However, sometimes it requires a pars plana vitrectomy to remove the hemorrhage because otherwise it may lead to retinal tears, retinal detachment, hemosiderosis bulbi and glaucoma, causing a further reduction in vision [1, 2]. Here, we report the early efficacy of a single intravitreal dobesilate injection in reducing vitreous hemorrhages caused by proliferative diabetic retinopathy.

## Case presentation

**Fig. 1:**
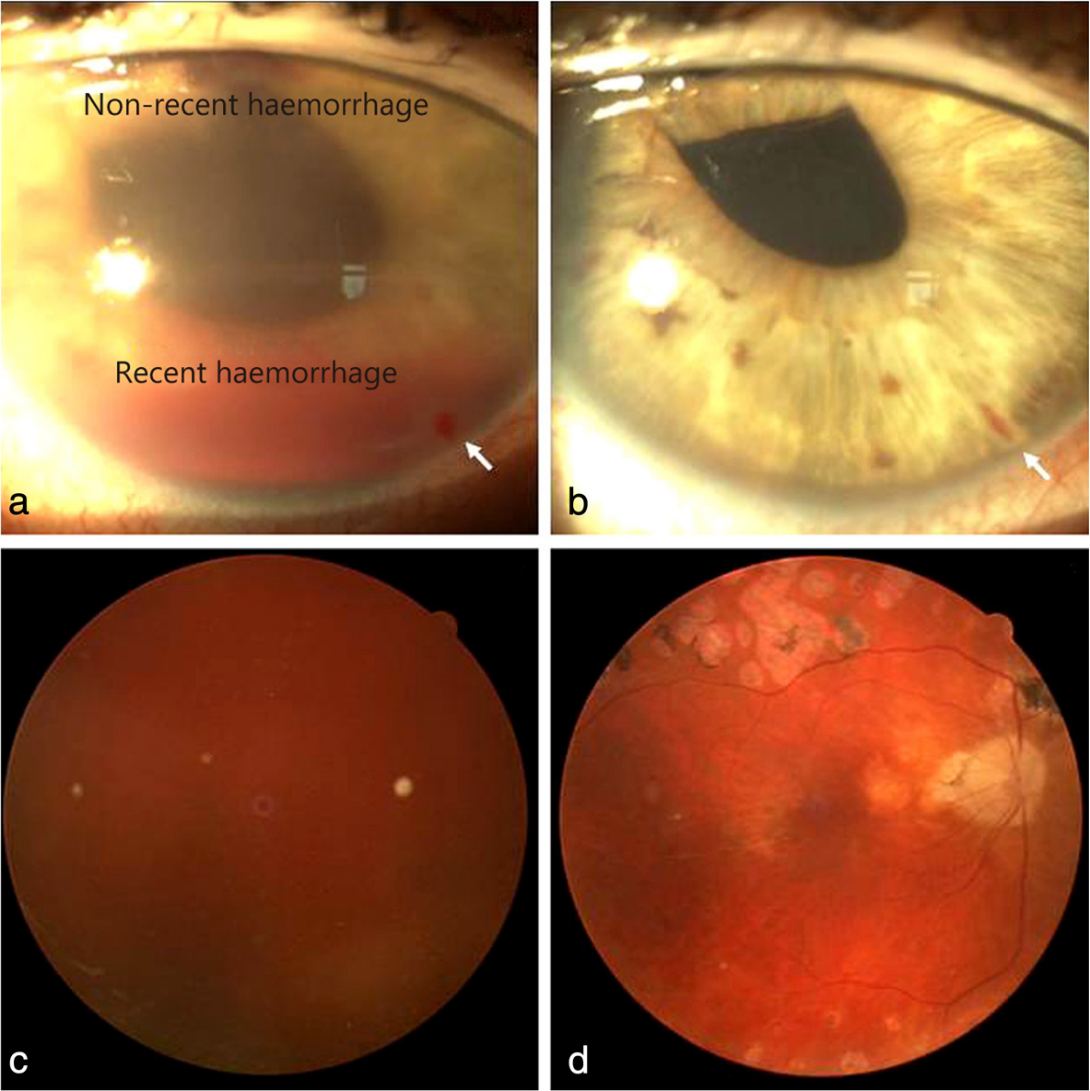
Efficacy of an intravitreal dobesilate injection to clear a vitreous hemorrhage in a diabetic patient. Photographs A and C were taken at presentation, and B and D were taken after 3 days of treatment. The non-recent and recent hemorrhages viewed at the presentation in the anterior segment (**a**) resolved after the treatment (**b**). At the presentation (**c**) the fundoscopy showed a blurred view of the retina caused by bleeding at the vitreous. After treatment (**d**), a clear view to the retina with residual blood was depicted. Note in B the abnormal appearance of the pupil due to luxation of the lens. The arrow indicates the paracentesis site

A 68-year-old Caucasian male with type 2 diabetes presented with one-month history of intense vision loss in his right eye. The patient did not seem had suffered any recent traumatic eye accident in the affected eye. The ophthalmic microscopic examination showed a pseudophakic right eye with both non-recent and recent hemorrhages in the anterior segment (Fig. 1a). A fundoscopic study revealed a massive vitreous hemorrhage (Fig. 1c). The intraocular pressure was 50 mmHg, which reduced to 14 mmHg after a paracentesis procedure.

### Treatment

After approval by the Institutional Review Board, the patient signed an informed consent form, which included a comprehensive description of the off-label use of dobesilate and the proposed procedure. The patient received an intravitreal solution of dobesilate (150 μl) under sterile conditions in his right eye according to the international guidelines for intravitreal injections [3]. Prophylactic topical antibiotics were given for 2 days postinjection. Dobesilate was administered as a 12.5 % solution of diethylammonium 2.5-dihydroxybencenesulfonate (etamsylate, Dicynone® Sanofi-Aventis. Paris. France). No ocular side effects were observed upon the administration of dobesilate or during the following days. Three days after the treatment, the hemorrhage in the anterior segment (Fig. 1b) had completely cleared. An obvious improvement was also appreciated in that of the vitreous cavity (Fig. 1d). Vision improvement was also observed. While the vision of the right eye was completely lost before the treatment was administered, the visual acuity determined by the Snellen chart became 0.4 three days after the treatment. The intraocular pressure remained normal.

### Discussion

A vitreous hemorrhage is a common disease that accompanies a wide variety of ophthalmological pathologies. The most common causes include proliferative diabetic retinopathy, vitreous detachment with or without retinal breaks, and trauma. Less common causes include vascular occlusive disease, retinal arterial aneurysms, hemoglobinopathies, neovascular age-related macular degeneration, and intraocular tumors. Hemorrhage into the vitreous gel results in rapid clot formation and is followed by a slow clearance of approximately 1 % per day [3]. The complications of persistant vitreous hemorrhages are hemosiderosis bulbi and glaucoma. The treatment option for non-clearing vitreous hemorrhage is a pars plana vitrectomy.

Dobesilate has been used for many years for the treatment of diabetic retinopathy. Its mechanism of action has not been established, but several possibilities have been proposed, including anti-inflammatory effects and inhibition of vascular endothelial growth factor (VEGF) [4, 5]. Although we cannot rule out the participation of these mechanisms, dobesilate clinical benefits for treatment of vitreous hemorrhage, probably should be largely attributed to its ability to inhibit the activity of fibroblast growth factor (FGF) that has been recently demonstrated [6]. FGF was the first inductor of vasculogenesis, proliferation of endothelial cells, and vascular permeability that was described [7]. Later, it was shown to be a broad-spectrum mitogen [8]. Recent data show that it should be better considered an inflammation-triggering and inflammation-sustaining protein, the mitogenic and permeability-inducing activities being manifestations of its inflammatory activity [9, 10]. The substantial increase in the permeability of the newly generated vessels has been attributed to FGF, which accumulates at high levels during diabetic retinopathy. In the case of hemorrhages, extravasated blood cells, such as monocyte-derived macrophages, also synthesize FGF [11]. FGF can also induce the expression of cyclooxygenase-2 (COX-2) and phospholipase A2, as well as the ensuing production of prostaglandins in endothelial cells, thereby creating a positive feedback loop that favors chronic bleeding and inflammation in a vicious cycle [12–16]. It has been reported that high levels of FGF induce structural changes in intestinal vessels, resulting in the development of lethal intestinal hemorrhages [17], and in FGF-induced corneal neovascularization, resulting in hyphemas [18]. We have previously shown that neovascular growth with its subsequent bleeding can be suppressed by inhibiting FGF [19]. Forty-four years ago, orally administered dobesilate was used to treat vitreous hemorrhage in diabetic patients with unclear efficacy [20]. This is likely due to the route of administration that does not allow dobesilate to reach adequate concentrations in the eye, as previously discussed [21]. Furthermore, we have recently reported that an intravitreal injection of dobesilate displayed clinical efficacy in a patient with a submacular hemorrhage secondary to age-related macular degeneration [22]. Here, we describe the dramatic clearance of a vitreous hemorrhage in a diabetic patient after a single intravitreal injection of dobesilate, which is a representative case of five patients who show similar therapeutic outcomes. Our study seems to be in full agreement with the use of etamsylate for the treatment of periventricular hemorrhages in infants with very low birth weight. Prophylactic treatment has also been shown to reduce intraventricular bleeding in babies of less than 34-weeks of gestation [23].

The data shown in this report seems support that direct application of dobesilate (a drug with a long history of clinical safety that was recently re-discovered as an FGF inhibitor) could serve as a promising new clinical application to temper the most important pathological effects of vitreous hemorrhages in proliferative diabetic retinopathy.

## Conclusion

An intravitreal injection of dobesilate for the treatment of vitreous hemorrhages related to proliferative diabetic retinopathy is associated with prompt morphologic and visual improvement and may offer benefits over the natural course of the disease.

## Consent

Written informed consent for publication of the clinical details and images was obtained from the patient.

## Abbreviations

FGF: Fibroblast growth factor
COX-2: Cyclooxygenase-2
